# Comparative transcriptomic analysis of mouse macrophages infected with live attenuated vaccine strains of *Mycobacterium tuberculosis*

**DOI:** 10.3389/fimmu.2025.1583439

**Published:** 2025-07-11

**Authors:** Raja Veerapandian, Barbara Yang, Areanna Carmona, Melina J. Sedano, Victoria Reid, Rodrigo Jimenez, Jessica Chacon, Chinnaswamy Jagannath, Enrique I. Ramos, Shrikanth S. Gadad, Subramanian Dhandayuthapani

**Affiliations:** ^1^ Center of Emphasis in Infectious Diseases, Department of Molecular and Translational Medicine, Paul L. Foster School of Medicine, Texas Tech University Health Sciences Center El Paso, El Paso, TX, United States; ^2^ Center of Emphasis in Cancer, Department of Molecular and Translational Medicine, Paul L. Foster School of Medicine, Texas Tech University Health Sciences Center El Paso, El Paso, TX, United States; ^3^ Frederick L. Francis School of Biomedical Sciences, Texas Tech University Health Sciences Center El Paso, El Paso, TX, United States; ^4^ Department of Medical Education, Paul L. Foster School of Medicine, Texas Tech University Health Sciences Center El Paso, El Paso, TX, United States; ^5^ Department of Pathology and Genomic Medicine, Houston Methodist Research Institute & Weill Cornell Medical College, Houston, TX, United States; ^6^ Department of Biology, University of Texas El Paso, El Paso, TX, United States

**Keywords:** Mtb-vaccines, BCG, mouse, macrophages, RNA-sequencing, transcripts, immune signaling, IL-17

## Abstract

The BCG vaccine has been used against tuberculosis (TB) for over a hundred years; however, it does not protect adults from pulmonary TB. To develop alternative vaccines against TB, we generated *Mycobacterium tuberculosis* H37Rv (Mtb)-derived vaccine strains by rationally deleting key virulent genes, resulting in single (*SKO; ΔfbpA*), double (DKO; *ΔfbpA-ΔsapM*), triple (TKO-D; *ΔfbpA-ΔsapM-ΔdosR* and TKO-Z; *ΔfbpA-ΔsapM-Δzmp1*), and quadruple (QKO; *ΔfbpA-ΔsapM-Δzmp1-dosR*) strains. To understand how macrophages, the host cells that defend against infection and process antigens for presentation to immune cells, respond to these vaccine strains, we performed transcriptomic analyses of mouse bone marrow-derived macrophages (BMDMs) infected with these strains. The transcriptomic data were compared with similar data obtained from macrophages infected with Mtb H37Rv and BCG. Our analyses revealed that genes associated with various immune and cell signaling pathways, such as NF-kappa B signaling, TNF signaling, cytokine-cytokine receptor interaction, chemokine signaling, hematopoietic cell lineage, Toll-like receptor signaling, IL-17 signaling, Th1 and Th2 cell differentiation, Th17 cell differentiation, and T cell receptor signaling were differentially expressed in BMDMs infected with our vaccine strains. Enhanced expression of cytokines and chemokines, including proinflammatory cytokines such as TNF-α, IL-6, GM-CSF, and IL-1, which are essential for the immune response against Mtb infection, was also observed in BMDMs infected with these strains. In particular, BMDMs infected with all vaccine strains exhibited a significant upregulation of genes associated with the IL-17 pathway. These results may indicate that our vaccine strains could induce a protective immune response against TB.

## Introduction

Tuberculosis (TB) is a deadly disease caused by an intracellular human pathogen, *Mycobacterium tuberculosis* (Mtb), which has coexisted with humans for approximately seventy thousand years (1, 2). According to the World Health Organization (WHO) report, nearly 10.8 million people were affected by TB, resulting in a mortality rate of 1.25 million in 2023 (3). Historically considered hereditary, TB was recognized as a contagious disease by Jean-Antoine Villemin in 1865, and Robert Koch identified the causative bacterium, Mtb, in 1882 (4). Selman Waksman developed Streptomycin, the first effective TB drug, earning him the Nobel Prize in 1952 (5). Conversely, the BCG vaccine developed by Albert Calmette and Camille Guérin remains the only licensed vaccine against TB. Unfortunately, BCG has not been regarded as an effective vaccine against TB because of the emergence of various sub-strains that produce differing levels of protective efficacy (6). The rise of multidrug-resistant TB (MDR-TB) has complicated treatment strategies even further, necessitating confirmation of bacterial infection and testing for antibiotic resistance. Alarmingly, only two out of five MDR-TB cases received treatment in 2022 (7).

In 2014, WHO launched the “End TB Strategy” (8) to significantly reduce the TB burden by 2035, emphasizing the critical role of vaccines. Various vaccine types, including live attenuated vaccines (LAV), subunit vaccines, viral vectored vaccines, DNA vaccines, whole-cell killed/inactivated vaccines, and recombinant protein-adjuvant formulations, have been developed and studied for TB prevention (9). Among these, LAV stands out for its ability to induce long-lasting immune responses, with BCG serving as a prime example. The BCG vaccine differs from the Mtb strain due to the deletion of various Mtb-specific open reading frames (ORFs) clustered in 16 genomic regions of difference (RD1–RD16) (10, 11). Mtb has also been modified to enhance its vaccine efficacy, particularly by knocking out the secretory proteins or secretory systems of mycobacteria (12).

As a first of its kind, we reported that the Mtb Δ*fbpA* strain protects mice against challenges similar to or better than BCG (13). Fibronectin-binding protein (FbpA; Rv3804c) is a secreted protein belonging to the Ag85 complex, which is highly conserved among species of the *Mycobacterium tuberculosis* complex. It has a mycolyltransferase enzyme function, catalyzing mycolic acid transfer during cell wall biogenesis (14). Gene disruption studies in Mtb demonstrated that FbpA is one of the key components necessary for intracellular survival (15). To enhance the vaccine efficacy, we additionally deleted the *sapM* gene in the Δ*fbpA* strain to create a double knockout (DKO) (16). The *sapM* gene (*Rv3310*) encodes the secreted acid phosphatase SapM, initially identified in Mtb (17). It interferes with the phagosome maturation by dephosphorylating PI-3 phosphate (18). Our DKO vaccine strain induces strong protection through enhanced antigen processing and the autophagy mechanism (19). To further enhance our DKO vaccine, we carefully deleted two additional genes, specifically *zmp1* (*Rv0198c*) and *dosR* (*Rv3133c*). Zmp1 is a ~75 kDa zinc metalloprotease secretory protein that plays a significant role in blocking phagosome maturation and impairing inflammasome activation, resulting in greater vaccine efficacy, (20, 21), whereas DosR is a dormancy survival regulator that collectively affects approximately fifty genes in the Mtb genome and is highly activated under microenvironmental conditions such as granulomas (22, 23). These new vaccine strains have shown increased immunogenicity (24), and efficacies against TB in animal models are being investigated.

This study follows up on our prior observation that a double-knockout (DKO) vaccine provides superior and longer-lasting protection compared to the BCG vaccine (19). In this study, we aimed to investigate the intricate molecular responses of macrophages to the Mtb-based live attenuated vaccines (LAVs) developed in our laboratory. Macrophages play a crucial role in defending against intracellular pathogens like Mtb and processing and presenting antigens to immune cells. An effective mycobacterial vaccine should induce key immune and cell signaling pathways that lead to effective antigen presentation and the subsequent pathogen clearance from the host. Thus, studying the molecular interactions between Mtb-derived vaccines and macrophages through RNA-seq analysis should provide important insights into vaccine efficacy. Although studies have documented genome-wide transcriptomic changes in human or mouse macrophages following Mtb infection (25–27), our study focuses on Mtb-derived vaccine strains for the first time. This approach has allowed us to identify the crucial immune and cell signaling pathways and profile the vital cytokines and chemokines for TB vaccines. Further, our findings underscore the importance of the IL-17 pathway regulated by LAV strains.

## Materials and methods

### Mtb strains and culture conditions

Wild-type and knockout Mtb strains were grown at 37°C in either Middlebrook 7H9 broth or 7H10 agar (BD Difco), both containing 0.05% Tween 80 (TW), 0.2% glycerol, and OADC (10%) enrichment. All mutant Mtb strains used in this study are a derivative of H37Rv. We published the single knockout, SKO (*ΔfbpA*) and double knockout, DKO (*ΔfbpA-ΔsapM*) strains used in this study earlier (13, 16). Triple knockouts such as ΔTKO-D (*ΔfbpA-ΔsapM-ΔdosR*), ΔTKO-Z (*ΔfbpA-ΔsapM*-*Δ*z*mp1*), and quadruple knockout, ΔQKO (*ΔfbpA-ΔsapM-Δzmp1-dosR*) strains were made on the DKO background, and their construction was briefly reported earlier (24) and will be published elsewhere (manuscript under preparation).

### Animals and ethics

C57BL/6J mice aged 4–6 weeks were purchased from Jackson Laboratories, Bar Harbor, ME. The mice received were housed with unlimited access to water and mouse chow and permitted to move without restraints within their cages at the Laboratory Animal Resource Center, Texas Tech University Health Sciences Center El Paso. The Institutional Animal Care and Use Committee (IACUC) of the Texas Tech University Health Sciences Center El Paso approved an animal protocol for this study (Protocol #17003).

### Isolation of bone marrow-derived macrophages

As described previously, BMDMs were extracted from wild-type C57BL/6 mice (28). Briefly, the BMDMs were cultured from the femurs and tibias of mice in DMEM medium (DMEM, 10% fetal bovine serum, 10 ng/ml of M-CSF) and incubated at 37°C in 5% CO_2_ for 7 days, with the addition of new medium containing M-CSF every 2–3 days.

### Infection of BMDMs with Mtb strains

Mtb wild-type H37Rv and all mycobacterial vaccine strains were cultured in 7H9 medium with appropriate antibiotics in roller bottles at 37°C for 5–7 days. Colony-forming units (CFUs) of the bacterial suspensions were determined and stored at -80°C until use. Before infection, bacteria were pelleted, washed with PBS, and dispersed using a 23G syringe to eliminate clumps. Mouse BMDMs (10^6^ cells/well) seeded in 6-well tissue culture plates (Corning, USA) were infected at a multiplicity of infection (MOI) of 1:5 in DMEM for 4 h to allow phagocytosis. Afterward, cells were washed thrice with D-PBS (Corning, USA) to remove extracellular bacteria and replaced with fresh DMEM containing 10% fetal bovine serum for further incubation at 24 and 72 h.

### RNA sequencing and data analysis

Mouse BMDMs (10^6^/well) were infected with the respective mycobacterial strains as described above for different time points (24 h and 72 h). After respective time points, total RNA was extracted from the infected BMDMs using the EZ-10 DNAaway RNA Mini-Preps Kit (Bio Basic, Canada) as described previously (28). RNA quantification was performed using Nanodrop (Thermo Scientific, USA). The quality of RNA was measured using TapeStation (Agilent Technologies 4200). The library preparation enriched for polyA RNA fraction was performed in house and RNA sequencing was performed at Novogene Corporation Inc. (Sacramento, CA, USA), as described elsewhere (29). Two biological replicates for each condition were performed. We employed web-based application Genialis to analyze RNA sequencing raw data using their “General RNA-Seq pipeline (featureCounts)” with default settings (Genialis, Inc., Boston, MA). RNA-Seq data were aligned using STAR aligner to the mouse transcriptome from Ensembl release version 109 with trimmed reads removing adapter sequences. Read counts were computed using featureCounts. Quality control metrics were determined, and the average quality per read was 36 (Phred score) (**Supplementary files - Table S1,S2**, **S2**). Principle component analysis (PCA) was generated within the Genialis RNA-seq pipeline visualization features. The differentially expressed genes (DEGs) were also computed using the Genialis built-in DESeq2 tool, defining the control samples of PBS or H37Rv and the case samples accordingly, and the filtering criteria for DEGs are FDR < 0.05 with log_2_ fold change greater than 1 for upregulated DEGs and less than 1 for downregulated DEGs. DEGs were presented in heatmaps, volcano plots, and Venn diagrams, using pheatmap, ggplot2, and Venn packages, respectively, in the R program. For downstream analysis of KEGG pathway analysis and Gene Ontology (GO) analysis for biological processes (BP), we queried the bioinformatic Database for Annotation, Visualization, and Integrated Discovery (DAVID) with default settings and plotted the top 30 KEGG pathways or BP based on ascending p-value as dot plots using ggplot2 in R, as described previously (28, 29).

### cDNA synthesis and qRT-PCR

The total RNA from infected BMDM was used to synthesize cDNA using the RevertAid First Strand cDNA Synthesis Kit (Thermo) according to the manufacturer’s protocol. Quantitative reverse transcriptase PCR (qRT-PCR) was performed using a LightCycler­^®^ 96 Instrument (Roche). PCR was performed using PowerTrack™ SYBR Green (Thermo Fisher Scientific) according to the manufacturer’s recommendations. Three biological replicates for each condition were performed. Primer details are given in the **Supplementary files** (**Supplementary Table S3**). The relative CT (ΔΔCT) method was used to quantify gene expression as described elsewhere (19). The expression levels of target genes were normalized to the house keeping gene, *actB* (ß- actin) with the H37Rv group set as the reference value 1 for comparison with all vaccine groups.

## Results

### Transcriptome analysis of mouse BMDMs infected with vaccine strains compared to uninfected cells

**Figure 1:**
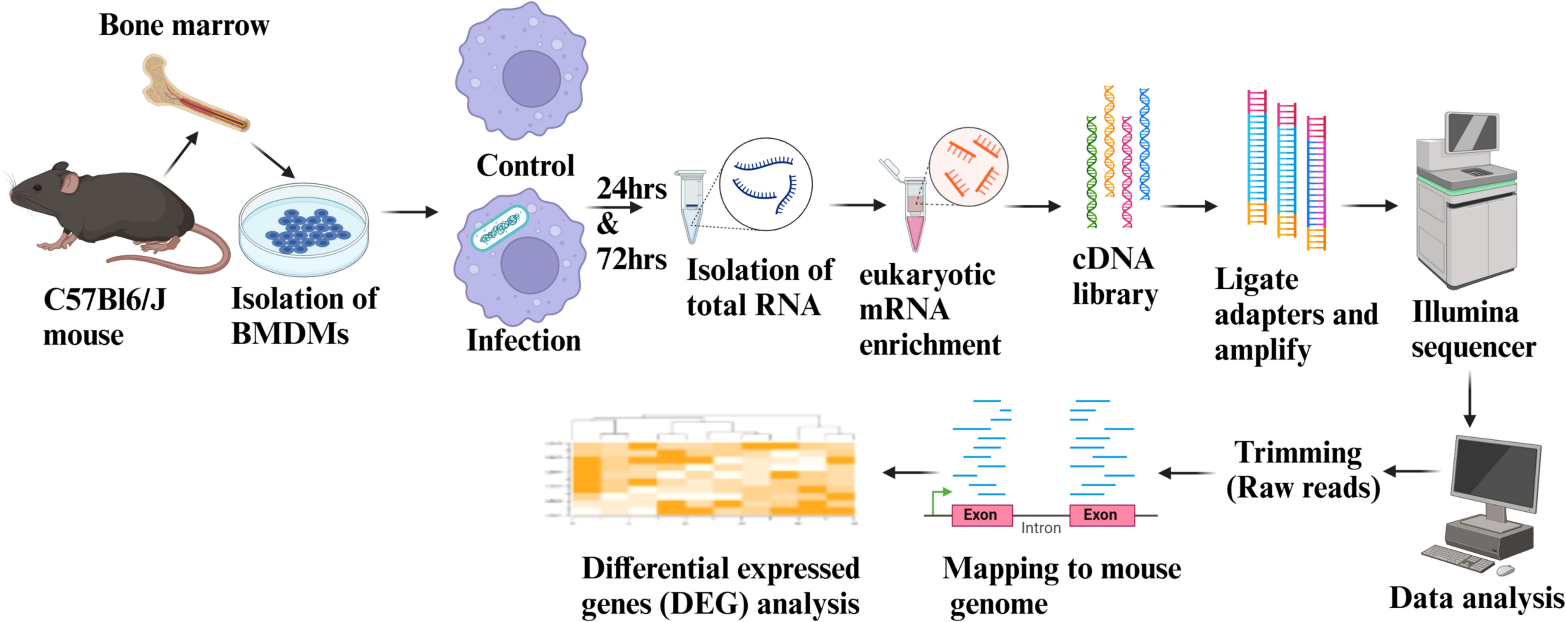
Schematics showing RNA-Seq workflow and data analysis. Fresh BMDMs were isolated from the female wild-type C57BL/6J mice and infected with respective vaccine strains or left uninfected. Following a 4-hour phagocytosis period, the BMDMs were washed with D-PBS and cultured in fresh DMEM supplemented with 10% fetal bovine serum for an additional 24 and 72 hours. RNA was isolated and subjected to eukaryotic mRNA enrichment at each time point. Subsequently, cDNA libraries were prepared, followed by adapter ligation and amplification for Illumina sequencing. The RNA-Seq data were aligned to the mouse transcriptome, and differential gene expression (DEG) analysis was performed. The figure was generated using BioRender.

To explore the variation in transcriptional signatures of mouse BMDMs infected with various Mtb vaccine strains, we conducted a genome-wide gene expression analysis using an RNA-sequencing platform (**Figure 1**). The performance of Principal Component Analysis (PCA) on the transcripts from mouse BMDMs clearly distinguished the infection groups from the PBS control at both 24 and 72 h time points. At 24 h post-infection, groups of transcripts with H37Rv background knockouts clustered almost entirely together, distinctly separating from those associated with BCG. By 72 h post-infection, almost all groups were distinctly separated, regardless of their H37Rv or BCG background (**Supplementary Figure S1**).

**Figure 2:**
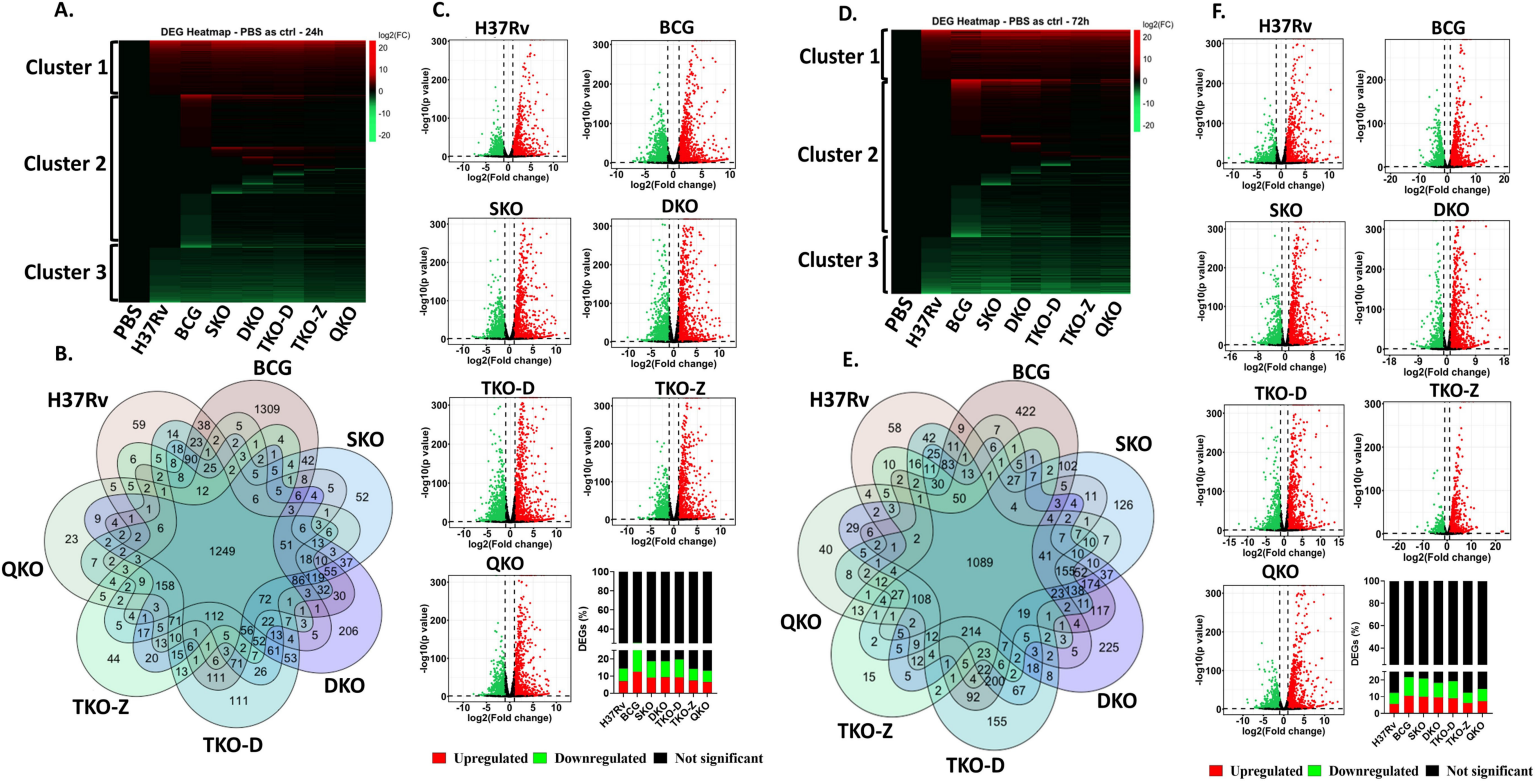
Transcriptome profiling of mouse BMDMs infected with vaccine strains in comparison to control. **(A, D)**, Heatmap of the differentially expressed genes at 24 h and 72 h; **(B, E)**, Venn diagram of the differentially expressed genes showing the number of overlapping and unique genes among groups at 24 h and 72 h; **(C, F)**, Volcano plot showing distribution of p values and log_2_ fold change of differentially expressed genes as green (downregulated), red (upregulated) and black (Not significant) at 24 h and 72 h with respective bar graphs in percentage.

For gene expression analysis, we considered fold change cut-off values of Log_2_ fold change >1.0 as upregulated and <-1.0 for downregulated genes (FDR < 0.05). Our transcriptome analysis identified more than 14,000 genes exhibiting expression among Mtb-infected mouse BMDMs compared to uninfected control (**Supplementary Data 1**, **2**). The heat map displayed a gene expression profile, showing a high disparity among infected groups compared to the PBS group, regardless of the time point (**Figures 2A, D**). Different clusters in the heat map indicate distinct modes of regulation, with cluster 1 being predominantly upregulated and cluster 3 downregulated across all vaccine groups. Interestingly, cluster 2 remains unchanged in the H37Rv wild-type group; however, there is a significant difference in this cluster among the vaccine groups. Notably, an additional change in cluster 2 of the vaccine groups is observed at the 72 h time point compared to 24 h. The Venn diagram illustrates both the unique and shared DEGs among the vaccine-infected groups. The total number of unique genes in the various vaccine-infected groups at 24 and 72 h post-infection are as follows: H37Rv (59 and 58), BCG (1309 and 422), SKO (52 and 126), DKO (206 and 225), TKO-D (111 and 155), TKO-Z (44 and 15), and QKO (23 and 40) (**Figures 2B, E**).

There are no significant percentage differences in differential gene expression between 24 and 72 h post-infection within the same group (**Figures 2C, F**). Compared to H37Rv background vaccine strains (~12.5-20.9%), BCG displays a higher percentage of DEGs at both time points (25.8 & 21.8%), while the H37Rv wildtype shows (14.4 & 12.4%) DEGs. Interestingly, the percentage of DEGs decreased successively as the gene deletion increased in vaccine strains such as SKO (18.7 &20.9%), DKO (18.8 &18.4%), TKO-D (19.8 &19.3%), TKO-Z (14.3 &12.5%), and QKO (13.2 &14.7%).

### Transcriptome analysis of mouse BMDMs infected with Mtb vaccine strains compared to H37Rv wild-type strain

**Figure 3:**
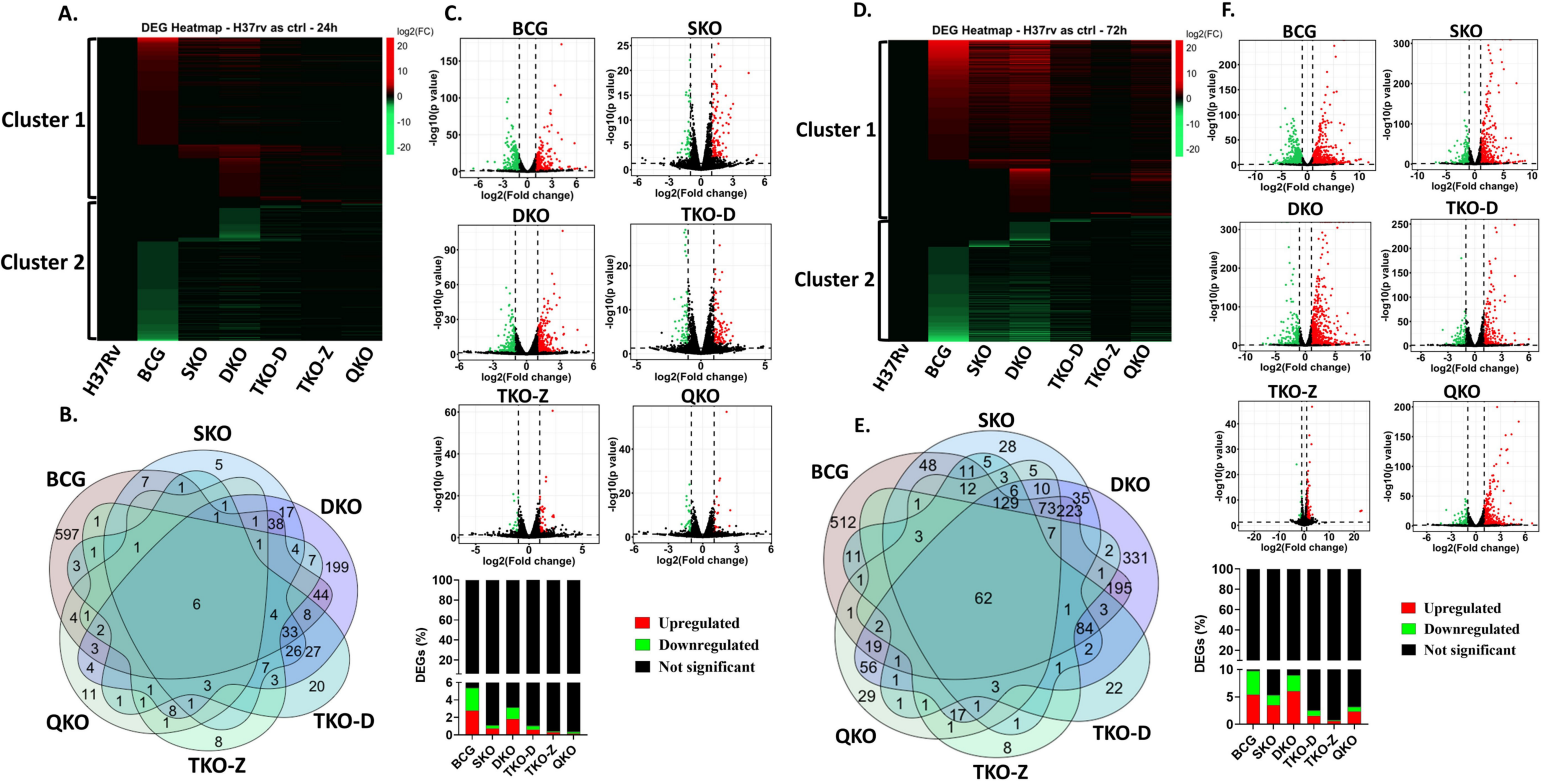
Transcriptome profiling of mouse BMDMs infected with vaccine strains in comparison to Mtb H37Rv. **(A, D)**, Heatmap of the differentially expressed genes at 24 h and 72 h; **(B, E)**, Venn diagram of the differentially expressed genes showing the number of overlapping and unique genes among groups at 24 h and 72 h; **(C, F)**, Volcano plot showing differentially expressed genes as green (downregulated), red (upregulated) and black (Not significant) at 24 h and 72 h with respective bar graphs in percentage.

As we observed differential regulation of genes within the vaccine groups, we proceeded to determine the number of DEGs in these groups compared to the H37Rv background (**Supplementary Data 3**, **4**). All our vaccine strains originate from the H37Rv background, where genes are sequentially deleted to create mutant strains. Thus, we compared all our vaccine strains, including BCG, with H37Rv. Unlike the previous comparison with uninfected, where three distinct clusters were observed, here we observed two distinct clusters. While minimal changes are noted in both clusters among the vaccine groups, with the exception of BCG and DKO, clusters 1 and 2 display a high number of upregulated and downregulated genes in the BCG group, along with only a few differentially regulated genes in the H37Rv background vaccine groups. Interestingly, certain alterations are observed in the H37Rv background vaccine groups in regions where the BCG group shows no changes. In contrast, the DKO group exhibits more drastic changes in gene expression in those regions (**Figures 3A, D**). The Venn diagram reveals a limited number of unique and shared genes among the vaccine groups. At 24 and 72 h post-infection, the number of unique genes in various vaccine-infected groups is as follows: BCG (597 and 512), SKO (5 and 28), DKO (199 and 331), TKO-D (20 and 22), TKO-Z (8 and 8), and QKO (11 and 29). Notably, BCG and DKO exhibit numerous unique DEGs, indicating distinct genetic responses (**Figures 3B, E**). According to the Volcano plot analysis, the percentage of DEGs at 24- and 72-h post-infection is as follows: BCG (5.4 &9.7%), SKO (1.1 &5.3%), DKO (3.1 & 9%), TKO-D (1 &2.6%), TKO-Z (0.4 &0.8%), and QKO (0.4 &3.2%). Greater percentage differences in differential gene expression are observed between 24- and 72 h post-infection within the same group, except for TKO-D and TKO-Z. Additionally, compared to H37Rv background vaccine strains (~0.4-9%), BCG exhibits a higher percentage of DEGs at both time points (5.4 & 9.7%) (**Figures 3C, F**).

### KEGG pathway analysis of mouse BMDMs infected with Mtb vaccine strains versus uninfected cells

KEGG pathway analysis was performed for all vaccine groups in comparison to the transcriptome of uninfected controls to identify pathways associated with mycobacterial infection. Several top enriched pathways were unique to the vaccine groups. Pathways such as graft-versus-host disease, allograft rejection, leishmaniasis, type I diabetes mellitus, TNF signaling, rheumatoid arthritis, inflammatory bowel disease, influenza A, NF-kappa B signaling, viral protein interaction with cytokines and cytokine receptors, Epstein-Barr virus infection, NOD-like receptor signaling, phagosome, cytokine-cytokine receptor interaction, Kaposi sarcoma-associated herpesvirus infection, measles, lipid and atherosclerosis, and COVID-19 were enriched with the upregulated DEGs of each condition across all vaccine groups (**Supplementary Figures S2**, **S3**). Conversely, pathways such as DNA replication, homologous recombination, cell cycle, Fanconi anemia, progesterone-mediated oocyte maturation, p53 signaling, oocyte meiosis, focal adhesion, cellular senescence, pathways in cancer, efferocytosis, ECM-receptor interaction, small cell lung cancer, motor proteins, PI3K-Akt signaling, Rap1 signaling, and MAPK signaling exhibited with downregulated DEGs across all vaccine groups (**Supplementary Figures S4**, **S5**).

### KEGG pathway analysis of mouse BMDMs infected with mycobacterial vaccine strains compared to H37Rv

**Figure 4:**
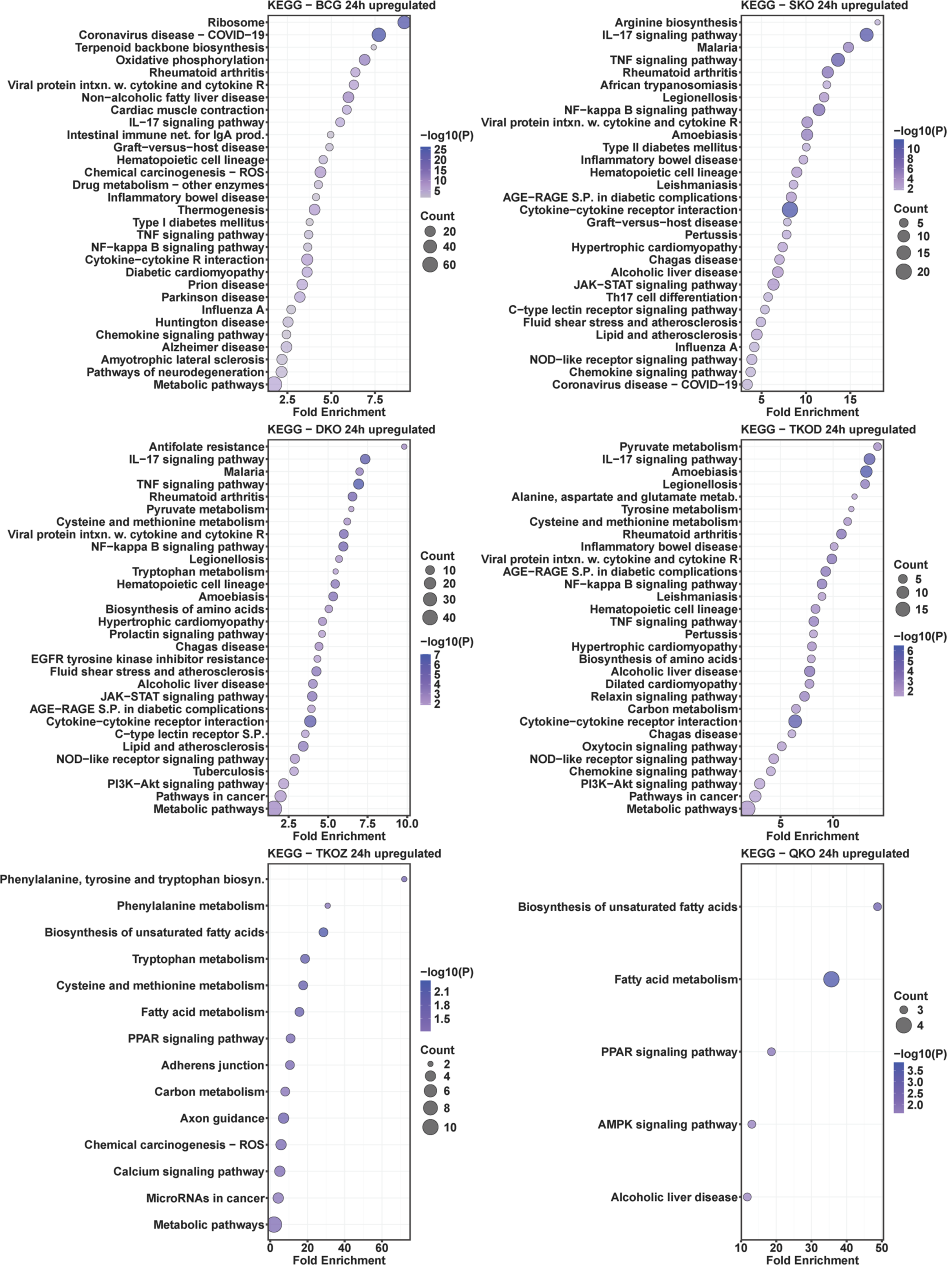
KEGG pathway analysis in upregulated differentially expressed transcripts of mouse BMDMs infected with vaccine strains versus H37Rv at 24 h post-infection. Dot plots illustrate the top 30 enriched pathways in upregulated DEGs in BCG, SKO, DKO, TKOD, TKOZ, and QKO compared to H37Rv control. Dot plots measure fold enrichment, where the dot size reflects the total number of genes in each pathway, and the gradient color indicates statistical significance expressed as −log_10_ (P).

**Figure 5:**
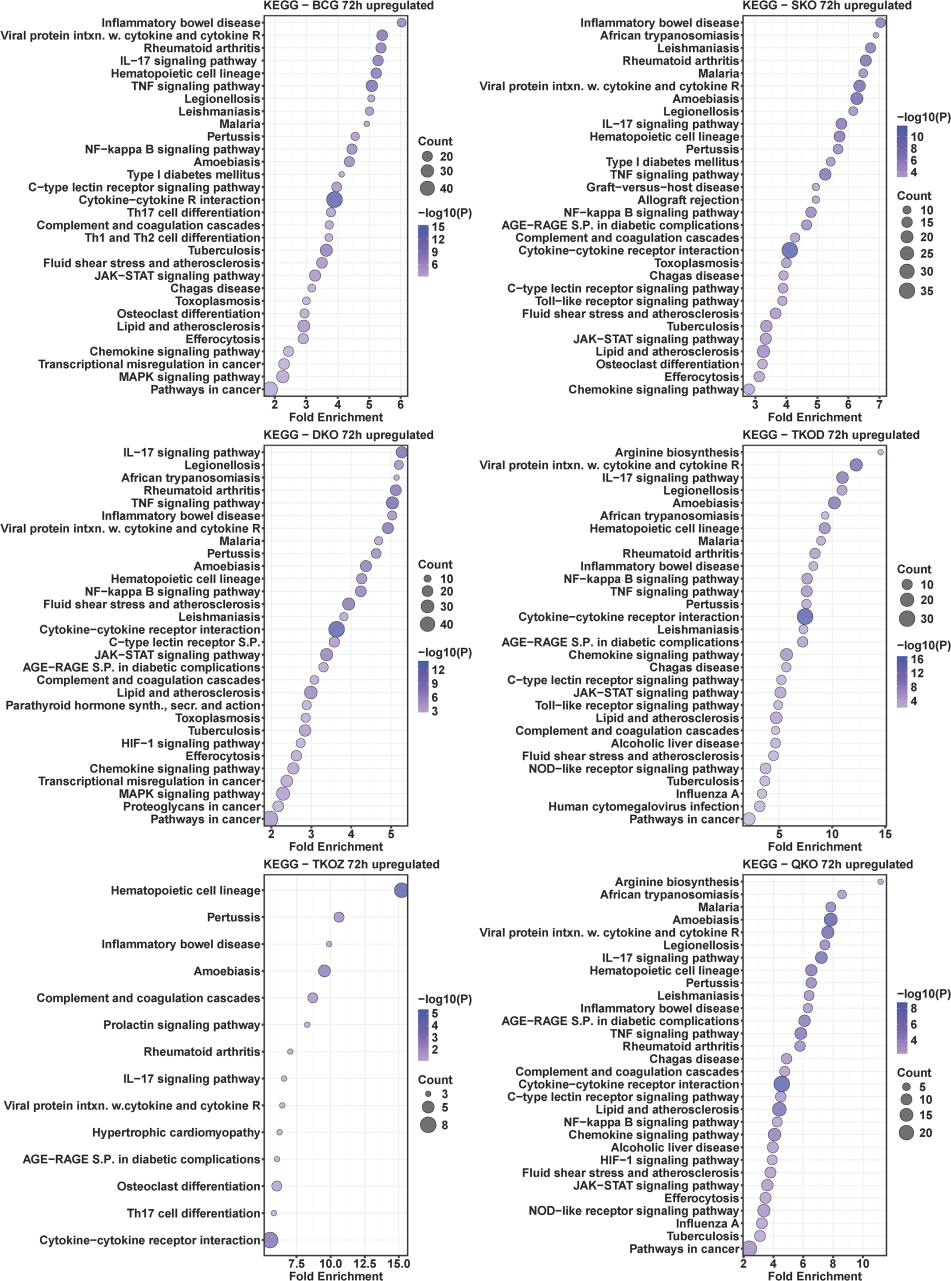
KEGG pathway analysis in upregulated differentially expressed transcripts of mouse BMDMs infected with vaccine strains versus H37Rv at 72 h post-infection. Dot plots illustrate the top 30 enriched pathways in upregulated DEGs in BCG, SKO, DKO, TKOD, TKOZ, and QKO compared to H37Rv control. Dot plots measure fold enrichment, where the dot size reflects the total number of genes in each pathway enriched, and the gradient color indicates statistical significance expressed as −log_10_ (P).

**Figure 6:**
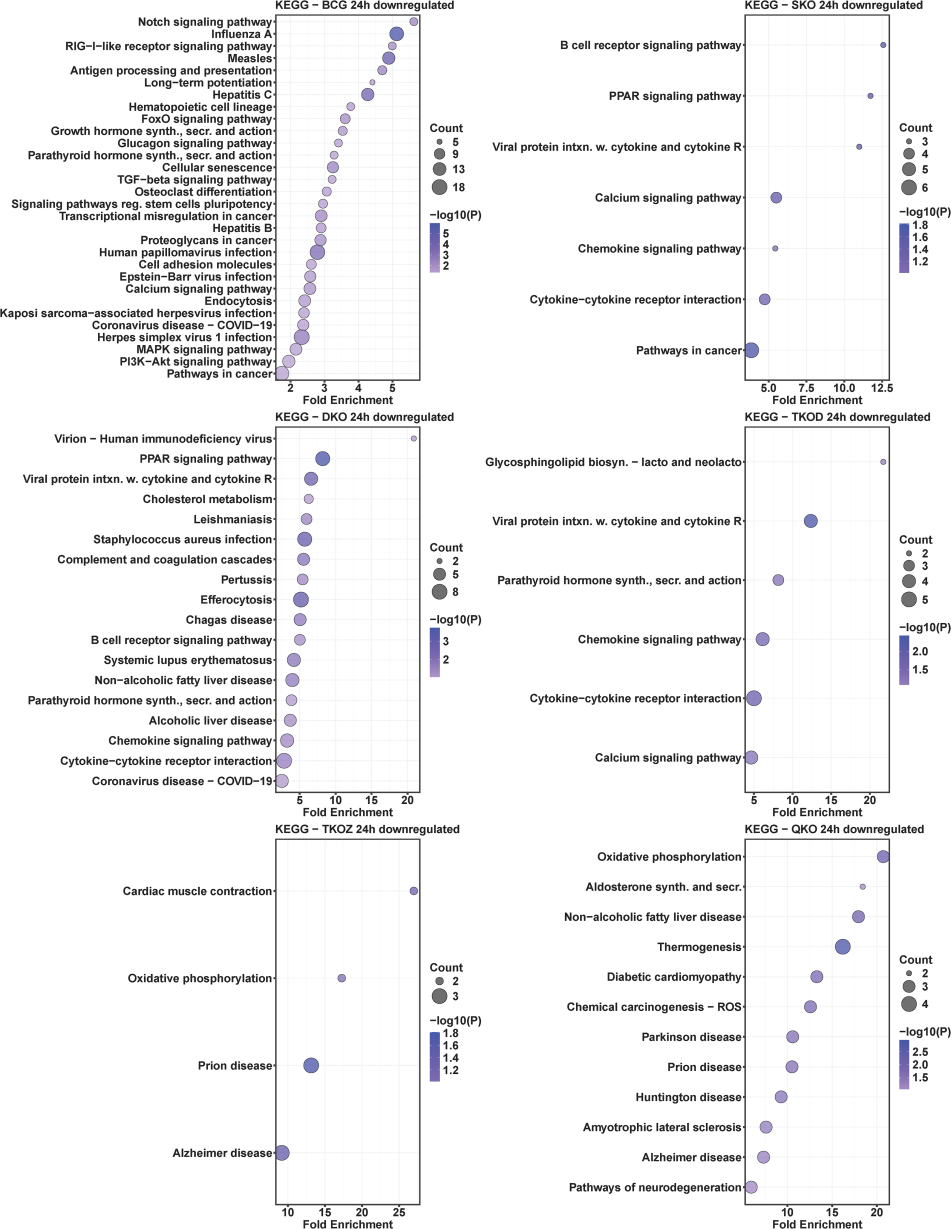
KEGG pathway analysis in downregulated differentially expressed transcripts of mouse BMDMs infected with vaccine strains versus H37Rv at 24 h post-infection. Dot plots illustrate the top 30 enriched pathways in downregulated DEGs in BCG, SKO, DKO, TKOD, TKOZ, and QKO compared to H37Rv control. Dot plots measure fold enrichment, where the dot size reflects the total number of genes in each category, and the gradient color indicates statistical significance expressed as −log_10_ (P).

**Figure 7:**
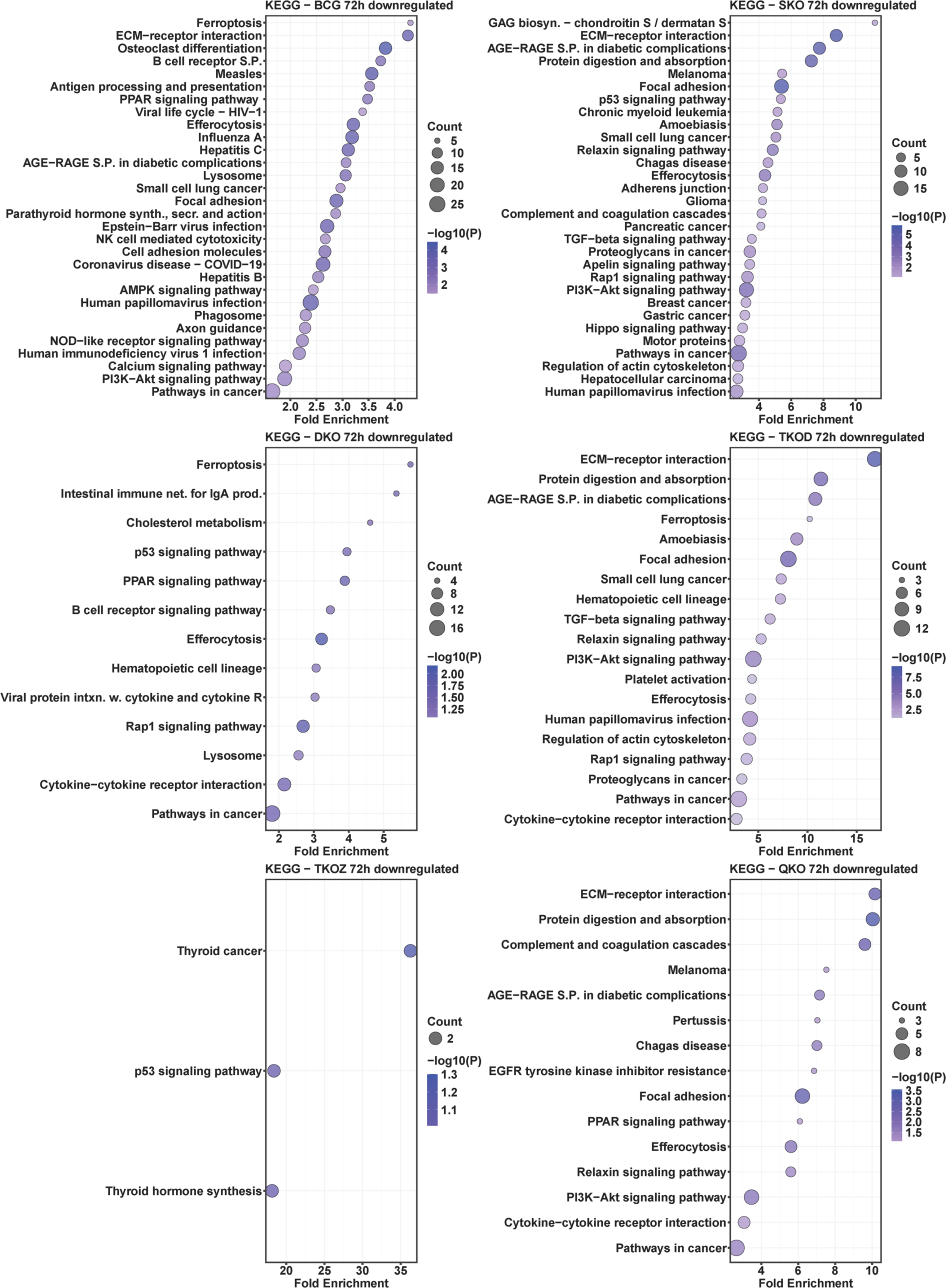
KEGG pathway analysis in downregulated differentially expressed transcripts of mouse BMDMs infected with vaccine strains versus H37Rv at 72 h post-infection. Dot plots illustrate the top 30 enriched pathways in downregulated DEGs in BCG, SKO, DKO, TKOD, TKOZ, and QKO compared to H37Rv control. Dot plots measure fold enrichment, where the dot size reflects the total number of genes in each category, and the gradient color indicates statistical significance expressed as −log_10_ (P).

To understand the roles of cellular pathways in the host’s response to mycobacterial infection, we performed a KEGG pathway enrichment analysis with the DEGs using Mtb H37Rv wild-type as the control. We identified multiple pathways that were uniquely and differentially dysregulated at both time points, with a similar pattern observed within the same groups across time points and between groups at each time point (**Figures 4**–**7**). Pathways including rheumatoid arthritis, viral protein interaction with cytokines and cytokine receptors, the IL-17 signaling pathway, hematopoietic cell lineage, and cytokine-cytokine receptor interaction were uniquely enriched with upregulated DEGs in all vaccine-infected macrophage groups compared to H37Rv (**Figure 5**). Notably, TKO-Z and QKO showed a delayed response in enriching those pathways in upregulated DEGs at 72 h. However, several pathways, such as the biosynthesis of unsaturated fatty acids, PPAR signaling, and fatty acid metabolism, exhibited overrepresentation in upregulatted DEGs (**Figure 4**) at 24 h. A delayed IL-17 response at 72 h was also noted in TKO-Z and QKO when compared to other vaccine groups (**Figure 5**).

Intriguingly, the pathways affected by downregulated DEGs varied across different vaccine groups. While BCG showed multiple pathways enriched in downregulated DEGs at both time points, our vaccine groups exhibited less pathways at 24 h (**Figure 6**) but pathways enriched increased in downregulated DEGs at 72 h (**Figure 7**). Specifically, TKO-Z had a few pathways, including cardiac muscle contraction, oxidative phosphorylation, prion disease, Alzheimer’s disease, thyroid cancer, p53 signaling, and thyroid hormone synthesis overrepresented in downregulated DEGs (**Figures 6**, **7**). Uniquely, the ferroptosis pathway, critical in mycobacterial infection, was enriched in both BCG and TKO-D downregulated DEGs at 72 h (**Figure 7**). Additionally, BCG, SKO, TKO-D, and QKO downregulated DEGs enriched the ECM-receptor interaction and focal adhesion pathways at 72 h, potentially limiting molecule translocation across barriers. Pathways such as protein digestion and absorption and PI3K-Akt signaling were enriched at 72 h in SKO, TKO-D, and QKO downregulated DEGs (**Figure 7**).

We also compared BCG with H37Rv, where pathways such as rheumatoid arthritis, viral protein interaction with cytokines and their receptors, IL-17 signaling pathway, hematopoietic cell lineage, inflammatory bowel disease, type 1 diabetes mellitus, TNF signaling pathway, NF-kappa B signaling pathway, cytokine-cytokine receptor interaction, and chemokine signaling pathway were similarly enriched with the upregulated DEGs in the BCG group at both time points (24 h and 72 h) (**Figures 4**, **5**). When observing the pathways enriched in downregulated DEGs, we noted pathways like influenza A, measles, antigen processing and presentation, hepatitis C, parathyroid hormone synthesis, secretion, and action, osteoclast differentiation, hepatitis B, human papillomavirus infection, cell adhesion molecules, Epstein-Barr virus infection, calcium signaling pathway, COVID-19, PI3K-Akt signaling pathway, and pathways in cancer were consistent at both time points (24 h and 72 h). However, multiple pathways were uniquely dysregulated at their respective time points (**Figures 6**, **7**).

Subsequently, we compared the pathways overrepresented in differentially regulated genes by BCG vaccine strains with those from Mtb-derived vaccines to assess how closely our newly developed Mtb-derived vaccines mimic BCG. Remarkably, similar KEGG pathways were enriched in our Mtb-derived vaccine strains compared to BCG in upregulated DEGs particularly at the 72 h time point (**Supplementary Figure S6**). At the 24 h time point, only a few pathways such as rheumatoid arthritis, viral protein interaction with cytokine and cytokine receptor, IL-17 signaling pathway, hematopoietic cell lineage, TNF signaling pathway, NF-kappa B signaling pathway, and cytokine-cytokine receptor interaction were uniquely enriched in upregulated DEGs in SKO, DKO, and TKO-D; almost no pathways matched with BCG for the TKO-Z and QKO groups. Interestingly, at 72 h, nearly all the pathways were uniquely enriched with the upregulated DEGs in our Mtb-derived vaccine groups compared to BCG. In examining the pathways enriched in downregulated DEGs, very few overlapped at 72 h, and almost none at 24 h (**Supplementary Figure S7**). Notably, the TKO-Z group did not exhibit any common pathways overrepresented in downregulated DEGs like BCG, in contrast to many that were enriched in upregulated DEGs in this group during 72 h. The-common KEGG pathways enriched in downregulated DEGs across all groups appeared primarily due to the SKO strain (**Supplementary Figure S7**).

### Gene ontology analysis of mouse BMDMs infected with mycobacterial vaccine strains compared to H37Rv

**Figure 8:**
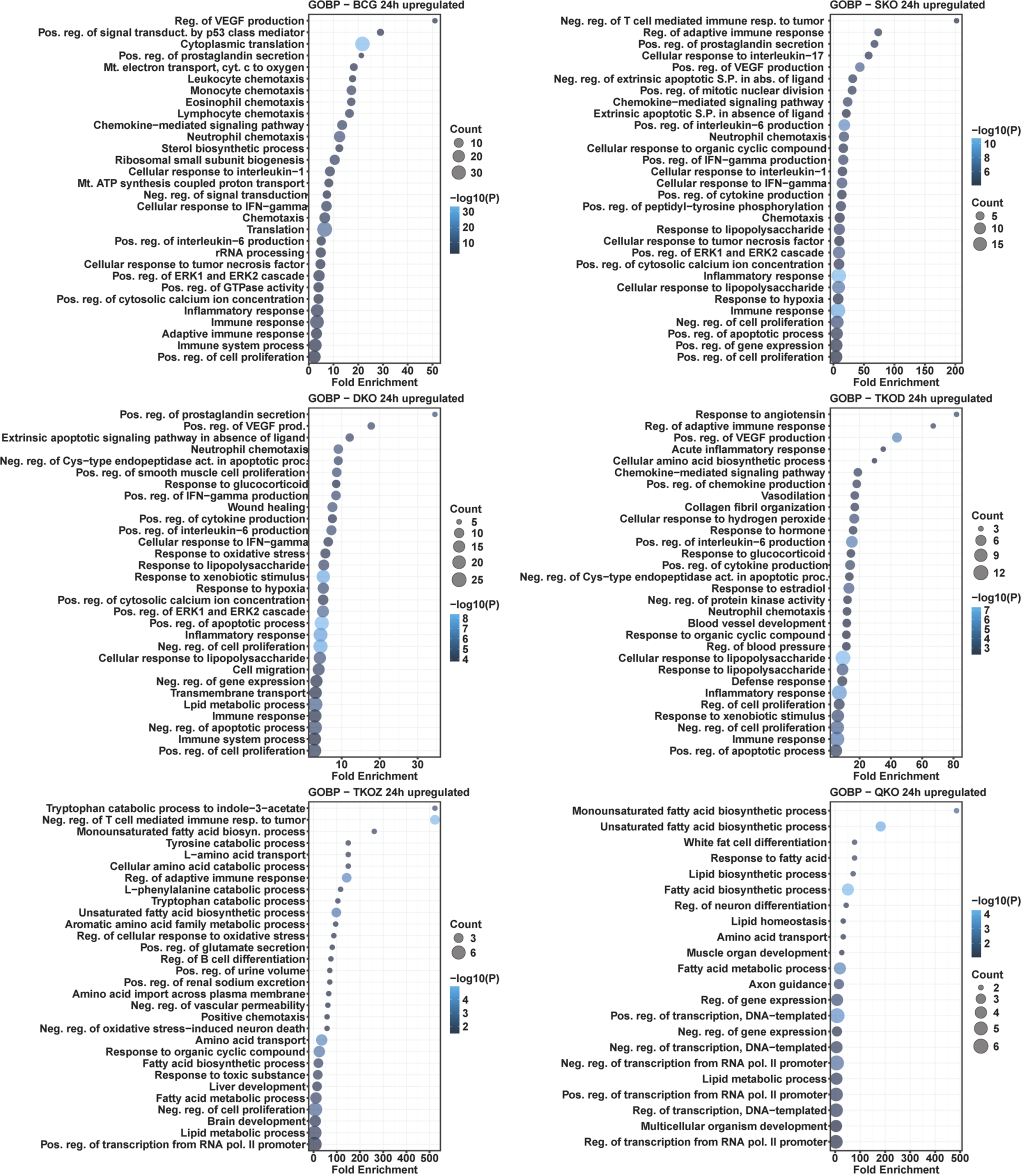
Gene ontology analysis for biological processes in the differentially upregulated transcripts of mouse BMDMs infected with vaccine strains versus H37Rv at 24 h post-infection. Dot plots illustrate the top 30 enriched biological processes in upregulated DEGs in BCG, SKO, DKO, TKOD, TKOZ, and QKO compared to H37Rv control. Dot plots measure fold enrichment, where dot size reflects the total number of genes in each biological process, and the gradient color indicates statistical significance expressed as −log_10_ (P).

**Figure 9:**
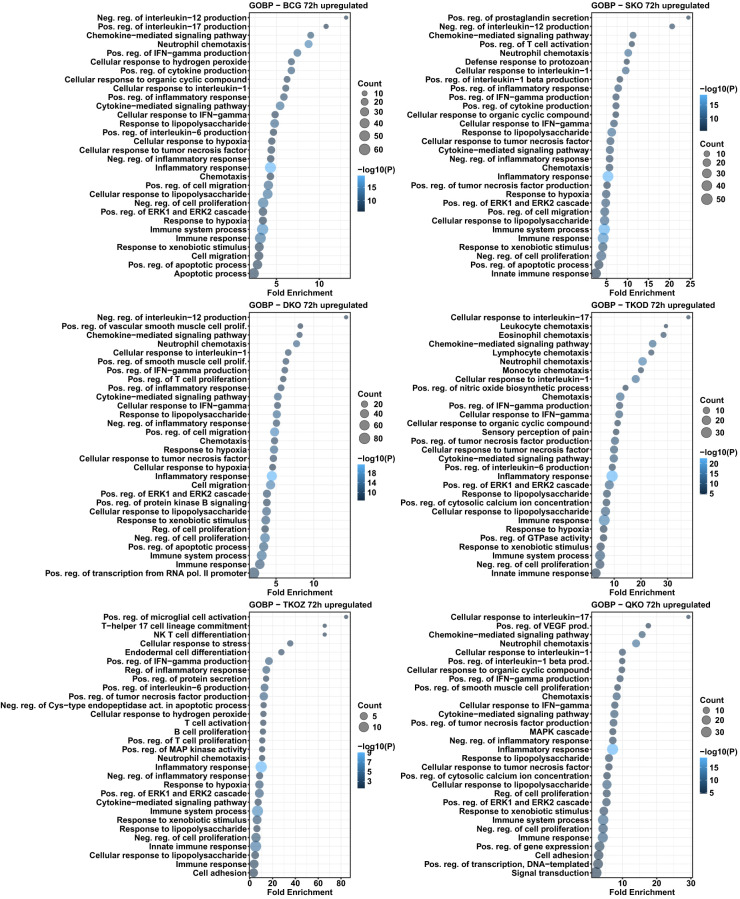
Gene ontology analysis for biological processes in the differentially upregulated transcripts of mouse BMDMs infected with vaccine strains versus H37Rv at 72 h post-infection. Dot plots illustrate the top 30 enriched biological processes in upregulated DEGs in BCG, SKO, DKO, TKOD, TKOZ, and QKO compared to H37Rv control. Dot plots measure fold enrichment, where the dot size reflects the total number of genes in each biological process, and the gradient color indicates statistical significance expressed as −log_10_ (P).

To investigate altered biological processes by DEGs, we performed GO analysis for biological processes for all vaccine groups compared to H37Rv. Biological processes, such as neutrophil chemotaxis, positive regulation of interleukin-6 production, inflammatory response, and immune response, were enriched with upregulated DEGs at the 24 h time point in the BCG, SKO, and TKO-D groups (**Figure 8**). Similar to the KEGG pathway analysis, the TKO-Z and QKO groups exhibited delayed enrichment of some common pathways in upregulated DEGs to other vaccine groups, primarily at the 72 h time point (**Figure 9**). At 72 h, biological processes, including neutrophil chemotaxis, positive regulation of interferon-gamma production, cytokine-mediated signaling pathway, response to lipopolysaccharide, inflammatory response, cellular response to lipopolysaccharide, negative regulation of cell proliferation, positive regulation of the ERK1 and ERK2 cascade, immune system process, immune response, and response to xenobiotic stimulus, were consistently overrepresented in the upregulated DEGs across all vaccine groups (**Figure 9**).

**Figure 10:**
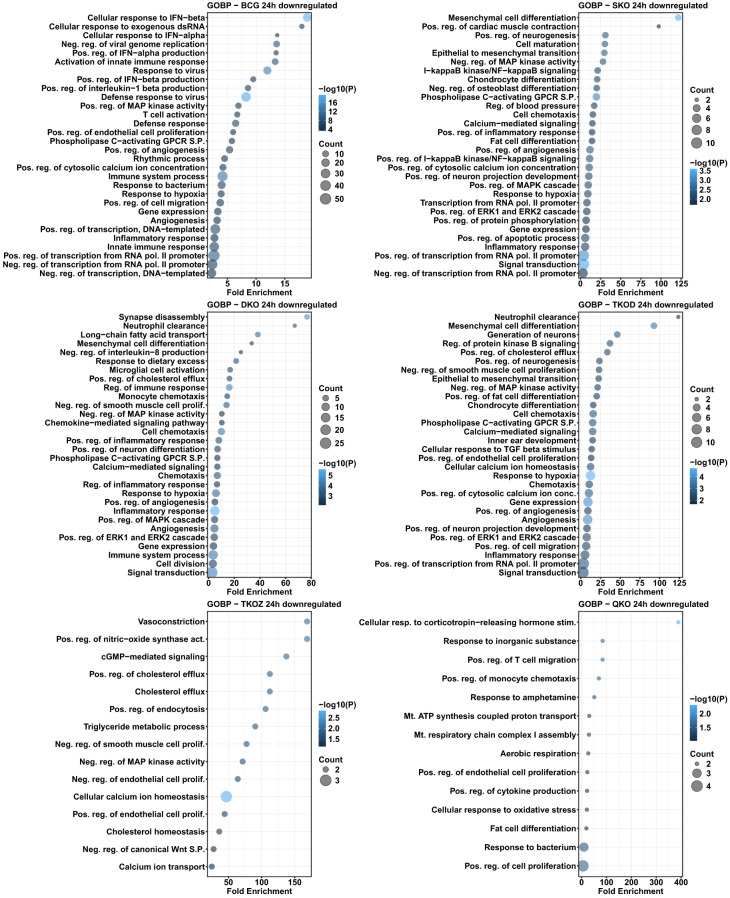
Gene ontology analysis for biological processes in the differentially downregulated transcripts of mouse BMDMs infected with vaccine strains versus H37Rv at 24 h post-infection. Dot plots illustrate the top 30 enriched biological processes of downregulated DEGs in BCG, SKO, DKO, TKOD, TKOZ, and QKO compared to H37Rv control. Dot plots measure fold enrichment, where the dot size reflects the total number of genes in each biological process, and the gradient color indicates statistical significance expressed as −log_10_ (P).

**Figure 11:**
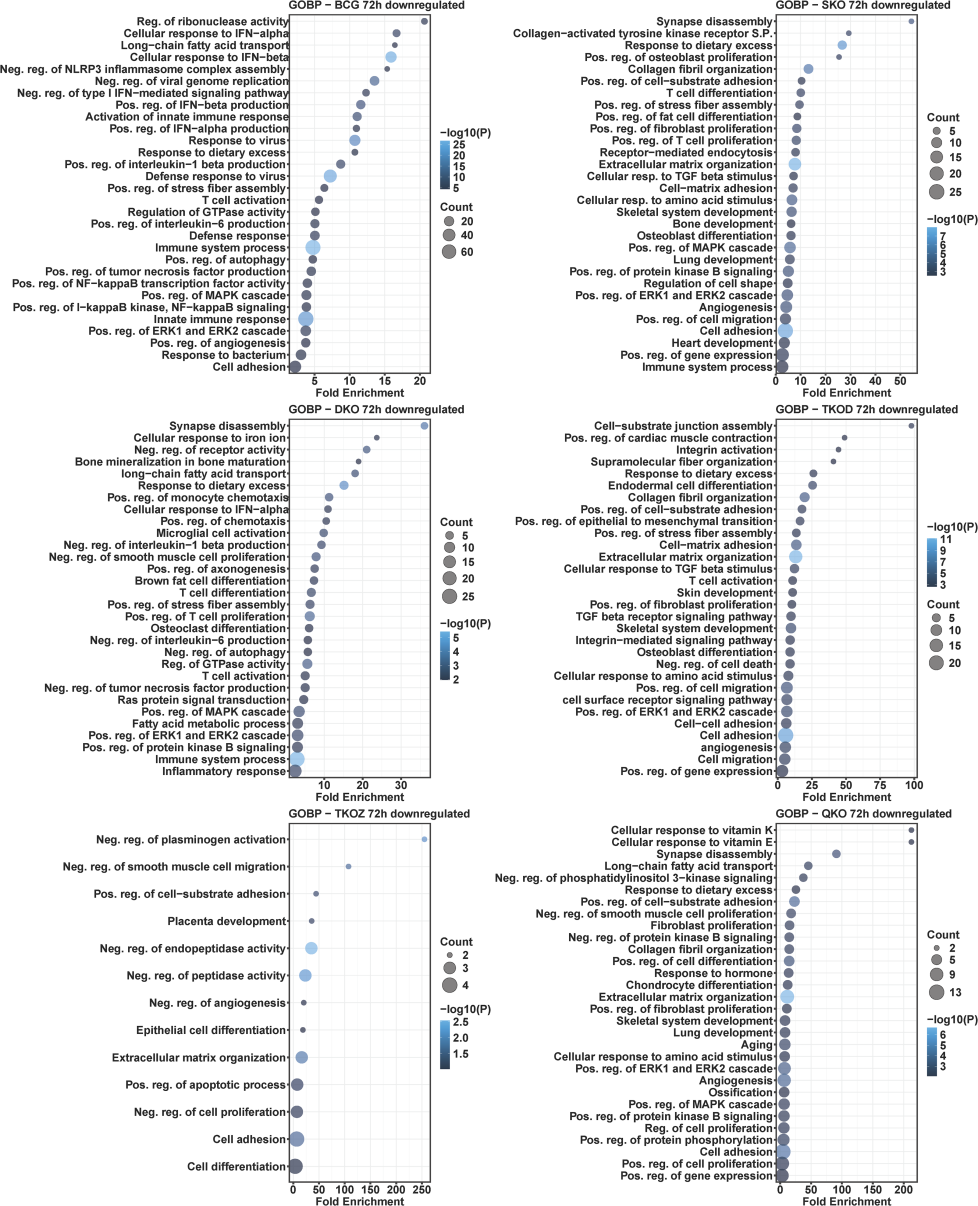
Gene ontology analysis for biological processes in the differentially downregulated transcripts of mouse BMDMs infected with vaccine strains versus H37Rv at 72 h post-infection. Dot plots illustrate the top 30 enriched biological processes in downregulated DEGs in BCG, SKO, DKO, TKOD, TKOZ, and QKO compared to H37Rv control. Dot plots measure fold enrichment, where the dot size reflects the total number of genes in each biological process, and the gradient color indicates statistical significance expressed as −log_10_ (P).

Similar to the KEGG pathway analysis, we observed a comparable pattern here, with a greater number of the enriched biological processes overlapping in the upregulated DEGs than in the downregulated DEGs. The TKO-Z group was particularly distinct, exhibiting overrepresented biological processes such as the positive regulation of endothelial cell proliferation and cell adhesion, in downregulated DEGs. Interestingly, most regulation of pathways appears to be linked to the deletion of *fbpA*, as indicated by the downregulated DEGs-enriched processes seen in the SKO group. These include the phospholipase C-activating G-protein coupled receptor signaling pathway, positive regulation of angiogenesis, positive regulation of cytosolic calcium ion concentration, response to hypoxia, gene expression, inflammatory response, positive regulation of transcription from the RNA polymerase II promoter, response to dietary excess, positive regulation of stress fiber assembly, immune system processes, positive regulation of the MAPK cascade, positive regulation of the ERK1 and ERK2 cascade, and cell adhesion (**Figures 10**, **11**).

### Key immune and cell signaling pathways differentially regulated among vaccine groups

KEGG pathway analysis was performed on immune and cell signaling pathways to investigate the mechanisms underlying vaccine-induced immune responses in macrophages infected with various vaccine strains (**Supplementary Data 5**, **6**). Importantly, gene deletions in our vaccine strains resulted in differential modulation of several signal transduction pathways, including the NF-kappa B signaling pathway (mmu04064) and TNF signaling pathway (mmu04668). The signaling molecule and interaction pathway, particularly Cytokine-cytokine receptor interaction (mmu04060), showed significant upregulation, while the ECM-receptor interaction pathway (mmu04512) demonstrated downregulation. Several cytokine and chemokine genes, including *tnf, il6, il1α, il1β, il1r1, il1r2, il12a, il12b, il23, cxcl1, cxcl2, cxcl3, ccl22, ccl2, ccl3, ccl4, ccl6, and ccl7*, were significantly upregulated across most vaccine groups. In contrast, genes such as *cxcr1, cxcr3, cxcl9, cxcl12, and ccl8* were downregulated in the majority of vaccine groups. Furthermore, as previously noted, the ECM-receptor interaction pathway was significantly enriched in downregulated DEGs in specific vaccine strain-infected BMDMs, including SKO, TKO-D, and QKO, at 72 h (**Figure 7**).

Key immune system pathways were also affected, including Hematopoietic cell lineage (mmu04640), Chemokine signaling pathway (mmu04062), Toll-like receptor signaling pathway (mmu04620), IL-17 signaling pathway (mmu04657), Th1 and Th2 cell differentiation (mmu04658), Th17 cell differentiation (mmu04659), and T cell receptor signaling pathway (mmu04660) (**Supplementary Data 5**, **6**). Notably, only a small number of genes in the B cell receptor signaling pathway (mmu04662) were differentially regulated across all vaccine strains, including BCG.

**Figure 12:**
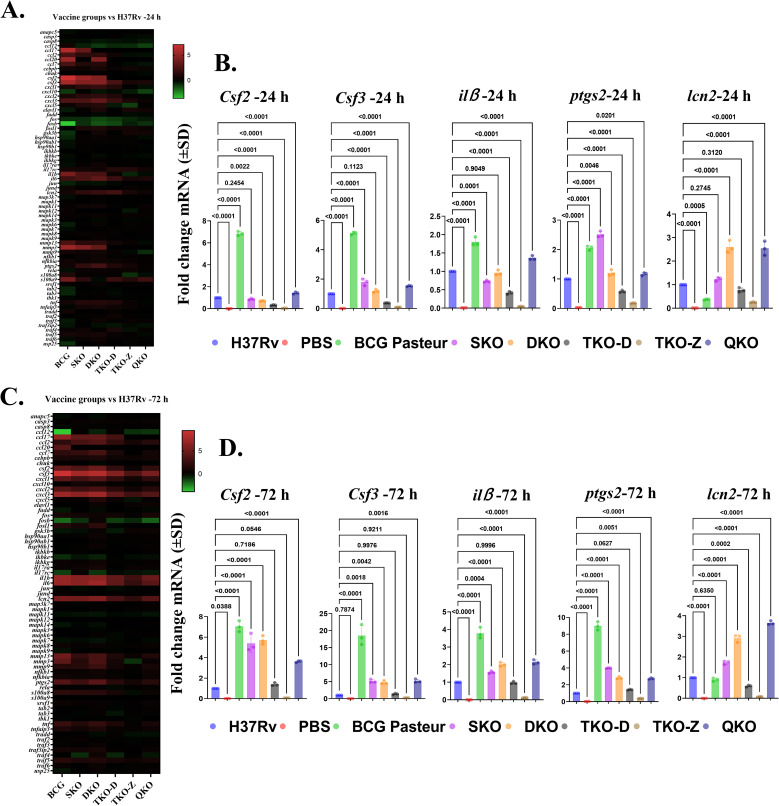
IL17 signaling pathway is differentially regulated in vaccine groups in comparison with H37Rv. **(A, C)** Heat map depicts the green–red gradient that reflects relative gene expression among vaccine groups at 24 h and 72 h. **(B, D)** Gene expression levels of Csf2, Csf3, ilß, ptgs2, and lcn2 in BMDMs infected with vaccine strains at 24 and 72 hours as determined by qRT-PCR. Data were analyzed by one-way ANOVA followed by Dunnett’s multiple comparisons test.

Among these pathways, the IL-17 signaling pathway exhibited the most pronounced differential regulation of DEGs across all vaccine groups. Heatmap analysis confirmed the list of genes with differential expression within the IL-17 signaling pathway (**Figure 12**). A few of the upregulated genes, such as *csf2, csf3, il1β, ptgs2, and lcn2*, were further confirmed by qRT-PCR, corroborating the transcriptome findings (**Figure 12**).

### Further confirmation of DEGs in vaccine-infected BMDMs using qRT-PCR

**Figure 13:**
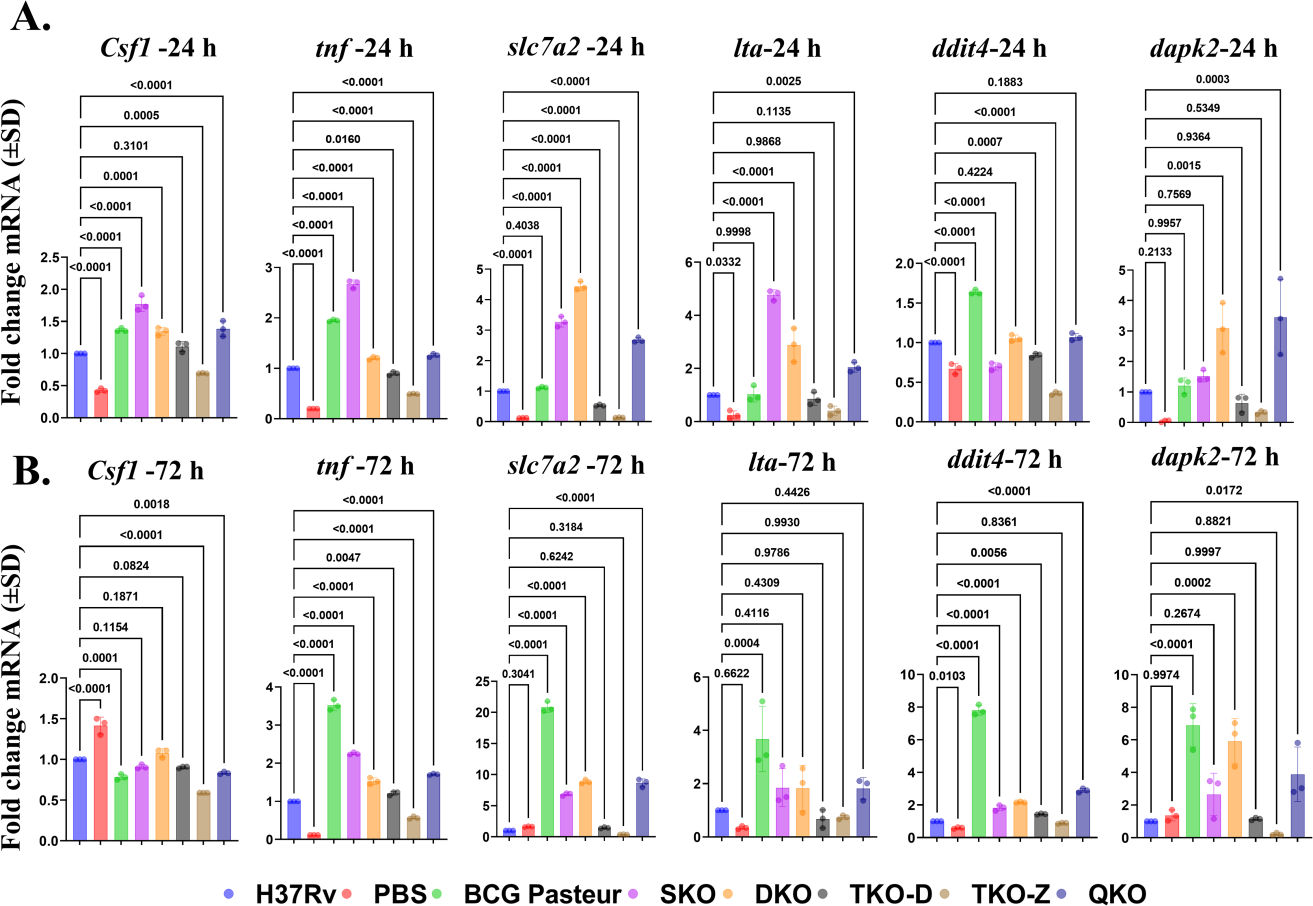
qPCR Validation of differentially regulated pathways in vaccine groups comparison with H37Rv. **(A, B)** Gene expression levels of Csf1, tnf, slc7a2, lta, ddit4, and dapk2 in vaccine strains infected BMDMs at 24 h and 72 h as determined by qRT-PCR. Data were analyzed by one-way ANOVA followed by Dunnett’s multiple comparisons test.

To further confirm the findings of our study, we performed qRT-PCR on macrophages infected with our vaccine strains. For this analysis, we randomly selected DEGs from various pathways. We examined genes such as *csf1, TNF, slc7a2, lta, ddit4*, and *dapk2* at the 24 h and 72 h time points (**Figure 13**). The results demonstrated strong alignment with the transcriptome data. Interestingly, we observed an increased expression of the solute carrier family 7-member 2 (*slc7a2*) gene across all our vaccine groups in both transcriptome and qPCR analyses (**Figure 13**). Notably, this gene is reported to be highly expressed in macrophages infected with avirulent Mtb strain H37Ra (25), indicating that our vaccine strains exhibit reduced virulence compared to the wild-type strain.

## Discussion

In this study, we performed genome-wide transcriptome analyses of mouse macrophages after infection with our Mtb-derived vaccine strains. We also included the BCG vaccine, as it is an established vaccine against TB. While multiple studies have reported transcriptome data of mouse macrophages infected with either BCG or Mtb, (25–27, 30, 31); our study focused on vaccine strains deficient in genes such as *fbpA*, *sapM*, *zmp1*, and *dosR*, either individually or in combination. Transcriptomic analysis of our vaccine-infected macrophages was conducted using a heatmap, volcano plot, and venn diagram, demonstrating a distinct difference in DEGs expressed among groups. The number of DEGs in BCG-infected macrophages was greater than in macrophages infected with other vaccine groups and Mtb H37Rv. This disparate response between BCG and our Mtb H37Rv-derived strains may be due to the deletion of several ORFs within RD1-RD16 regions in BCG.

KEGG pathway analysis of DEGs identified several immune pathways that are implicated in vaccine-infected macrophages. Some prominent pathways include infections with intracellular bacteria, as well as immune, viral, cancer, and disease-related components. Type I interferon-related pathways, such as cytosolic DNA-sensing, NOD-like receptor signaling, NF-kappa B signaling, and C-type lectin receptor signaling, were significantly enriched with upregulated DEGs in our vaccine strains compared to the naïve group, similar to the previous reports (30, 32, 33). Also, consistent with earlier reports, TNF signaling was enriched with upregulated DEGs in all vaccine groups (25, 34). Notably, pathways such as the cell cycle, DNA replication, p53 signaling, progesterone-mediated oocyte maturation, focal adhesion, and efferocytosis were enriched with downregulated DEGs in all vaccine groups, with some of the pathways aligning with earlier findings (30, 35).

Significant variations in upregulated DEGs-enriched pathways were noted across the vaccine groups between 24 and 72 h. Notably, TKO-Z and QKO exhibited a few pathways unique to other vaccine strains at 24 h, but by 72 h, they aligned with strains like SKO, DKO, and TKO-D. This change in pathways in TKO-Z and QKO may result from the deletion of zmp1. The zmp1 gene encodes a crucial enzyme for *M. tuberculosis* pathogenicity, playing various roles such as inhibiting phagosome maturation, suppressing inflammasome activation, mediating necrosis, and providing protection in guinea pig models (20, 21, 36). Interestingly, intracellular bacterial pathways, including legionellosis, leishmaniasis, and tuberculosis, were not enriched with upregulated DEGs in the TKO-Z group compared to H37Rv at either time point. However, these pathways were significantly enriched in the TKO-Z group when compared to the uninfected control. Due to the lack of transcriptome data for a *zmp1* mutant infected macrophages, direct comparisons with previous findings cannot be made. In comparison to H37Rv, the upregulation of genes in the H37Rv-derived vaccine groups ranged from approximately 0.2 -6.1%, while the downregulated DEGs showed minimal changes, with only about 0.1-2.9% of genes being downregulated. This highlights the need for transcriptome data from Mtb-derived vaccine candidates currently in clinical trials for a more comprehensive comparison. Similar to BCG, pathways such as ECM receptor interaction, efferocytosis, focal adhesion, and PI3K-Akt signaling were enriched with downregulated DEGs in some vaccine groups in our study.

Despite developing numerous vaccines against TB, we still lack exact knowledge of the immune correlates of protection (CoPs) for TB. However, data from animal and human studies provide insights into the immune cells that may be crucial for controlling TB, including Th1, Th17, CD8+ T cells, B cells, tissue-resident memory T cells, trained immunity, and tissue-resident alveolar macrophages (37). Interestingly, genes from the major immune and cell signaling pathways like Cytokine-cytokine receptor interaction, Chemokine signaling, NF-kappa B signaling pathway, Toll-like receptor signaling, IL-17 signaling, Th1 and Th2 cell differentiation, Th17 cell differentiation, T cell receptor signaling, and TNF signaling pathway were differentially regulated in our vaccine strains compared to wildtype H37Rv. Numerous vaccine studies have underscored the crucial role of T cell-mediated protection against Mtb infection (19, 38–41) and the limited significance of B cell-mediated responses in TB vaccines (42). Similarly, our vaccines mainly boost T cell-mediated immune pathways instead of B cell receptor signaling pathways. These findings offer a hopeful perspective for the development of more effective TB vaccines.

Cytokines and chemokines are essential in coordinating the immune response to mycobacterial infection (43). TB vaccine candidates like VPM1002 and MTBVAC have shown increased cytokine responses (44, 45). Our vaccine strains similarly showed different cytokine and chemokine expression profiles. Pro-inflammatory cytokines, including TNF-α, IL-6, GM-CSF, and IL-1, are crucial for the immune response against Mtb infection and play a vital role in host survival (46–48). Consistent with previous findings, our vaccine-infected macrophages exhibit increased expression of TNF-α, IL-6, and GM-CSF. Furthermore, our vaccine strains induce higher levels of IL-1 family cytokines, including IL-1α, IL-1β, IL-1R1, and IL-1R2. Chemokines such as CXCL1, CXCL2, and CXCL3 promote the recruitment of neutrophils and natural killer cells, while CCL3 and CCL4 aid in T-cell recruitment. Additionally, CCL7 is vital for recruiting monocytes, dendritic cells, T cells, and natural killer cells (49). Notably, our vaccine strains show strong expression of these chemokines, highlighting their potential immunomodulatory role effects.

Emerging evidence underscores the essential role of IL-17 in TB control across various species, including mice (50, 51), non-human primates (52, 53), and humans (54). Initially, IL-17 was thought to primarily mediate responses against extracellular pathogens rather than intracellular bacteria like Mtb. However, recent findings underscore its essential role in TB control. Studies have revealed that IL-17 levels are significantly lower in individuals who progress to active TB compared to non-progressors (55). Reports indicate that CD4+ T cells producing IL-17 are primarily localized in the lungs compared to TNF-α and IL-2. Furthermore, administering exogenous IL-17 in human granuloma models has shown effectiveness in controlling Mtb (56). Moreover, IL-17 has been identified as essential in mice for providing early protective immunity against Mtb HN878 infection (51). Mice that lack IL-17 receptors show reduced long-term control of Mtb infection (57). In our study, IL-17 signaling was significantly upregulated across all vaccine strains, including SKO, DKO, TKO-D, TKO-Z, and QKO. Gene ontology analysis revealed upregulated cellular responses to IL-17 in TKO-D and QKO, T-helper 17 cell lineage commitment in TKO-Z, and positive regulation of IL-17 production in the BCG vaccine group. These findings underscore the pivotal role of IL-17 in TB control and the effectiveness of our vaccine strains in eliciting an appropriate immune response.

Recent studies have reported that the upregulation of *slc7a2* in macrophages plays a critical role in controlling the intracellular survival of Mtb (25). Notably, *slc7a2* expression is higher in macrophages infected with the avirulent strain H37Ra compared to the virulent H37Rv strain. Consistent with these findings, our DEGs analysis revealed increased expression of *slc7a2* transcripts in our vaccine strains compared to the wild-type H37Rv, a result further validated through qPCR. While safety studies in SCID mice are still required to establish the safety profile of our vaccine strains, these findings suggest an improved safety profile for the vaccine strains used in this study.

One of the major limitations of the present study is that the comparative transcriptomic analysis was performed under *in vitro* conditions and not *in vivo*. While our experimental design provides us with the controlled environment to study BMDMs’ responses after infection with our vaccine strains, it lacks the *in vivo* conditions like interactions with other cell types, location-specific cell signals, etc. However, our study offers valuable comparative transcriptomic analysis datasets among our vaccine strains along with BCG, which offer insights that help enhance our understanding. This study focuses exclusively on comparing the transcriptomes of vaccine strains derived from the H37Rv Mtb strain. Further research is required to understand the relationship between the immune and cell signaling pathways activated by these vaccines and their actual protective efficacy. Moreover, this study emphasizes the importance of performing comparative transcriptomic analyses for vaccine candidates such as VPM1002 and MTBVAC, currently undergoing clinical trials, to gain deeper insights into the host immune response. Simultaneously, we recognize the importance of ‘decoy’ immune responses in TB infection (58). While certain host immune responses may appear promising, they indeed support the pathogen by promoting its persistence within the host. Thus, we strongly underscore the importance of performing protection studies in animal models and correlating these immune responses to actual protection, rather than relying solely on the statement that heightened proinflammatory cytokine production alone is beneficial. Overall, our study provides a thorough comparative transcriptome analysis of Mtb-derived vaccine strains alongside BCG, highlighting key immune pathways that play a crucial role in modulating immune and cell signaling events in the fight against the Mtb pathogen.

## Data Availability

The new RNA-seq genomic datasets presented here can be accessed from the NCBI Gene Expression Omnibus (GEO) database (http://www.ncbi.nlm.nih.gov/geo/) with the following accession number: GSE290804.
